# Bioinformatics identification of mitochondrial dynamics-related potential biomarkers in kawasaki disease and prediction of potential regulatory correlations

**DOI:** 10.3389/fcvm.2026.1856834

**Published:** 2026-07-28

**Authors:** Tingting Hu, Shaoyong Lin, Rongrong Yang, Xiaofeng Guo

**Affiliations:** Department of Cardiology, Fujian Children's Hospital (Fujian Branch of Shanghai Children's Medical Center), College of Clinical Medicine for Obstetrics & Gynecology and Pediatrics, Fujian Medical University, Fuzhou, Fujian, China

**Keywords:** immune infiltration analysis, kawasaki disease, machine learning, mitochondrial dynamic, molecular regulatory network

## Abstract

**Background:**

Kawasaki disease (KD) is a systemic vasculitis. Mitochondria was found to promote the activation of NLRP3 inflammatory vesicles, which have been shown to be a key driver of vascular disease. And there are few relevant reports of mitochondrial dynamic (MD) in KD. This study aimed to distinguish the potential biomarkers related to MD in KD and supply ideas for the intervention and treatment of KD. Currently, functional experimental verification in this field is lacking, and this study preliminarily explores their potential correlations through bioinformatics analysis.

**Methods:**

Differential expression analysis, machine learning, and expression validation were employed to identify potential biomarkers. Subsequently, analyses like enrichment analysis, immune infiltration analysis, and molecular regulatory network were applied to probe the underlying mechanisms of potential biomarkers.

**Results:**

RAP2C, DPM2, and DDX59 were identified as potential biomarkers associated with MD in KD and were mainly involved in inflammation-related pathways. Notably, 13 differential immune cells were gained in KD and control groups, 9 of which showed strong correlation with potential biomarkers, suggesting a relationship between potential biomarkers and the immune microenvironment in KD. For example, DPM2 had the positive relationship with CD8 T cells, while DDX59 had the negative relationship with CD8 T cells. Afterwards, molecular regulatory networks of potential biomarkers were constructed, specifically, they shared 5 common microRNAs (miRNAs) and 3 common transcription factors (TFs). Ultimately, potential biomarkers had 3 common targeting drugs [valproic acid, benzo(a)pyrene, and acetaminophen]; notably, benzo(a)pyrene was excluded due to carcinogenicity, which supplied the basis for targeting potential biomarkers to treat KD.

**Conclusion:**

The potential biomarkers (RAP2C, DPM2, and DDX59) related to MD in KD were acquired. These findings provide preliminary insights for future drug repurposing studies targeting KD and offer bioinformatic evidence for a potential link between mitochondrial dynamics and the immune microenvironment in KD.

## 1 Introduction

Kawasaki disease (KD) is an acute, self-limiting vasculitis with an unclear pathogenesis, also known as mucocutaneous lymph node syndrome. It predominantly occurs in children younger than 5 years old and has become one of the primary causes of acquired heart disease in developed nations (1). The major clinical manifestations of KD consist of sustained high fever, bilateral non-exudative conjunctival hyperemia, oral and labial mucosal changes, non-specific skin rash, peripheral extremity abnormalities, and non-suppurative cervical lymphadenopathy (2). The most severe complication of KD is coronary artery lesions (CALs), which can progress to coronary artery aneurysm (CAA), thrombosis, myocardial infarction, and even sudden death in critical cases (3). Studies have indicated that approximately 25% of KD patients are not diagnosed and treated promptly, resulting in CALs and potential sudden death; however, the precise pathophysiology remains incompletely understood (4). Some patients present with atypical clinical manifestations, coupled with non-specific laboratory findings, serve only an auxiliary diagnostic role, thereby potentially leading to the condition being underdiagnosed, delayed treatment, and eventual severe complications (5, 6). Accumulating evidence indicates that the pathogenesis of KD is linked to intricate factors including infection, genetic predisposition, and immune reactions. It is mainly characterized by abnormal immune activation and excessive pro-inflammatory cytokine secretion, resulting in substantial patient heterogeneity and diagnostic difficulties (6, 7). Therefore, to better guide the clinical diagnosis and management of KD, it is imperative to investigate its pathogenesis. Identifying potential biomarkers influencing KD progression or developing new therapeutic strategies is of particular importance.

As highly dynamic cellular structures, mitochondria function as core platforms for energy metabolism and intracellular signaling, and are involved in pivotal biological processes including ATP synthesis, reactive oxygen species (ROS) production, oxidative phosphorylation, and maintenance of calcium homeostasis (8).In eukaryotic cells, mitochondria continuously undergo dynamic processes including fission, fusion, mitophagy, and transport, collectively referred to as Mitochondrial Dynamics (MD), which are crucial for maintaining mitochondrial morphology, function, and cellular homeostasis (9, 10). Dysregulation of MD has been strongly implicated in the development and progression of multiple diseases, such as neurodegenerative, cardiovascular, metabolic disorders and malignancies (11, 12). Studies have found elevated levels of mitochondrial DNA (mtDNA) in the serum of patients with KD, and mtDNA exacerbates acute-phase inflammatory responses by activating the cGAS-STING signaling pathway (13). Moreover, mitochondrial dysfunction modulates immune cell function, facilitates NLRP3 inflammasome activation, and induces excessive pro-inflammatory mediator secretion, ultimately resulting in vascular endothelial damage (14). Of note, MD, including fission, fusion and mitophagy, are crucial for preserving mitochondrial homeostasis and controlling cellular function. Emerging evidence suggests that MD imbalance contributes to the pathogenesis of diverse vascular inflammatory disorders. In vascular endothelial cells, inflammatory stimuli can promote excessive mitochondrial fission, resulting in mitochondrial dysfunction, excessive ROS production, and impaired endothelial function, ultimately exacerbating vascular inflammation and injury (15). Given that KD pathophysiology is linked to infection, genetic predisposition and immune dysregulation, together with emerging evidence supporting mitochondrial dysfunction, we hypothesized that disrupted MD may contribute to KD pathogenesis. Given the pivotal role of MD in various diseases, investigation of mitochondrial dynamic genes (MDGs) in KD may identify innovative diagnostic and therapeutic targets.

In summary, to comprehensively elucidate the potential mechanisms of MDGs in KD, this study employed transcriptomic analysis to identify MDG-related differentially expressed genes (DEGs) in KD patients. Integrated with machine learning approaches, MDG-associated potential biomarkers were identified and validated. We explored the effects and underlying mechanisms of these potential biomarkers in KD pathogenesis, providing a potential theoretical basis for further exploring prevention and therapeutic strategies.This study is a bioinformatics prediction research, only conducting correlation analysis based on public database data, and subsequent *in vitro* and *in vivo* functional experiments are required to further verify the conclusions

## 2 Materials and methods

### 2.1 Data sources

The datasets were all acquired from the Gene Expression Omnibus (GEO) database. Specifically, GSE73461 (platform: GPL10558) included data from 77 KD and 55 healthy whole blood samples from individuals, which was named the training set. Meanwhile, GSE68004 (platform: GPL10558) included data from 76 KD and 37 healthy whole blood samples from individuals, which were named as the validation set. Then, according to the literature (16), 23 mitochondrial dynamics genes (MDGs) were acquired from the MitoCarta database. These genes included MFN1, MFN2, OPA1, DNM1L (DRP1), MFF, FIS1, FIS2, MIEF1, MIEF2, SLC25A46, DNM2, OMA1, YME1L1, PARL, HTRA2, LETM1, OPA3, MIRO1, MIRO2, TRAK1, TRAK2, GDAP1 (Supplementary Table S1).

### 2.2 Differential expression analysis and WGCNA

Two GEO datasets, GSE73461 (training set) and GSE68004 (validation set), were strictly selected for this study following three core criteria: both datasets were generated on the same microarray platform GPL10558, eliminating cross-platform technical heterogeneity; both contained whole blood samples from KD patients and age/sex-matched healthy controls, perfectly matching the study objective of exploring KD-related mitochondrial dynamics; both had complete clinical grouping information and high-quality expression data, ensuring the reliability of subsequent analyses. Raw expression matrices of the two datasets were independently preprocessed with uniform quality control (QC) procedures: negative expression values were corrected, and log2-transformation was performed to stabilize gene expression variance (17); probes were mapped to official gene symbols, and non-protein-coding genes, low-expression probes (expression value < 1 in >50% samples), and duplicate probes (retaining the probe with the highest mean expression per gene) were removed. To avoid confounding batch effects between datasets, GSE73461 and GSE68004 were analyzed independently without merging—GSE73461 was used for differential expression analysis and potential biomarker identification, while GSE68004 served as an independent validation set.

Differentially expressed genes (DEGs) between KD and control groups in dataset GSE73461 were identified using the limma [v 3.54.0 (17)] (|log2FC| > 0.5, *p* < 0.05). Firstly, according to the expression of genes in the GSE73461, the ssGSEA algorithm of the GSVA (v 1.50.0) (18) was applied to calculate ssGSEA scores for MD in KD and control samples. Afterwards, the Wilcoxon test was applied to analyse the differences in ssGSEA scores (*p* < 0.05). Prior to the WGCNA analysis, to minimize the interference of low-variability genes in the construction of the co-expression network, genes were pre-filtered based on the median absolute deviation (MAD), retaining only those with a MAD value above the 25th percentile and an absolute value greater than 0.001. Next, the GoodSamplesGenes function in the WGCNA package (v 1.72.5) (19) was used to check for missing data in genes and samples within the expression matrix. Sample outlier status was assessed based on Euclidean distance and fully connected hierarchical clustering, and samples that did not meet the quality standards were excluded. To construct a scale-free co-expression network, the pickSoftThreshold function was used to evaluate the network's topological fit index at different soft thresholds within a preset range of power exponents. A scale-free topological fit index (R^2^) of 0.80 was set as the primary criterion, while also considering average connectivity; the soft threshold meeting these conditions was selected accordingly. Subsequently, the blockwiseModules function was used for module identification. The network type was set to signed, the correlation metric to Pearson, deepSplit to 2, the minimum number of genes per module to 100, and the module merging height threshold to 0.25, thereby completing the partitioning of co-expression modules. The Hmisc (v 5.13) (20) was employed to calculate Spearman cor between MDGs scores and modular features in the gene module, and the results were presented via the pheatmap (v1.0.12) (|cor| > 0.3, *p* < 0.05). Modules with the strongest correlation were chosen as key modules (|MM| > 0.8 and |GS| > 0.2).

### 2.3 GO and KEGG of candidate genes

After, the ggvenn (v 0.1.9) (21) was applied to gain candidate genes from the intersection of DEGs and key module genes. Subsequently, to further understand the pathways involved in the candidate genes, the clusterProfiler (v 4.7.1.3) (22) was applied to conduct GO and KEGG analyses of candidate genes (*p* < 0.05).

### 2.4 Machine learning

So as to acquire the feature genes, the glmnet [v 4.1.4 zz (23) was applied to conduct LASSO regression analysis. We used the expression matrix of the candidate genes as the input variable and the group status (KD vs. control) as the response variable. The model type was set to binomial (family=“binomial”], the regularization parameter alpha was set to 1, and the random seed was fixed at 18 to ensure reproducibility of the results. We iterated through a sequence of values for the penalty parameter *λ* using 10-fold cross-validation (nfolds = 10), and selected *λ*.min (the value corresponding to the minimum cross-validation bias) as the optimal *λ* value, thereby identifying the LASSO feature genes. At the same time, the SVM-RFE algorithm of the caret (v 6.0.93) (24) was applied to gain the SVM-RFE-feature gene. The random seed was fixed at 2, and a Support Vector Machine with a radial basis kernel (svmRadial) was selected as the base learner. Using the rfeControl function to set the cross-validation strategy to 10-fold cross-validation (method = “cv”, numbe*r* = 10) and specifying the feature subset sizes as all integers from 1 to the total number of candidate genes [sizes = 1:ncol(candidate_matrix)]. Finally, the feature subset yielding the highest average accuracy across the 10-fold cross-validation is selected as the SVM-RFE feature genes. Subsequently, the VennDiagram (v 1.73) (21) was applied to acquire common feature genes by taking the intersection of two gene sets. Furthermore, to assess the ability of the feature genes to discriminate between KD and control samples, based on GSE73461 and GSE68004, the pROC (v 1.18.0) (25) was employed to draw the ROC curve on the feature genes (AUC > 0.7). Ultimately, expression analysis was performed on genes with an AUC greater than 0.7 in both the GSE73461 and GSE68004 datasets. Specifically, genes that exhibited expression differences between the KD group and the control group and showed consistent expression trends across both datasets were selected as potential biomarkers for subsequent analysis.

### 2.5 Construction of a nomogram

To investigate whether the potential biomarkers could predict the prevalence of KD, based on the GSE73461, the rms (v 6.5.0) (26) was applied to construct a nomogram. Moreover, the ggDCA (v 1.2) was applied to plot a DCA to further validate the clinical potential of the nomogram.

### 2.6 GSEA

In particular, according to the GSE73461, the psych (v 2.2.5) (27) was applied to analyze the associations between each potential biomarker and other genes. The background gene set was gained from the MSigDB (c2.all.v7.2.symbols.gmt), the clusterProfiler (v 4.7.1.3) was applied to conduct the GSEA (|NES| > 1, *p* < 0.05). In addition, we performed Spearman's correlation analysis using the stats package (version 4.2.2) to examine the relationships between the potential biomarkers and core MD genes (DNM1L, MFN1, MFN2, OPA1, FIS1, and MFF) (*P* < 0.05).

### 2.7 Immune infiltration analysis

To comprehend changes in the immune microenvironment in the KD patients, based on the 22 immune cell types (28), the CIBERSORT algorithm (29) was applied to analyze the abundance of immune cells in the GSE73461. Meanwhile, we excluded samples that did not differ between KD and control samples (*p* < 0.05). Afterwards, the Wilcoxon test was applied to acquire differential immune cells between KD and control samples (*p* < 0.05). Additionally, the psych (v2.2.5) was employed to analyze Spearman correlations between key immune cells and potential biomarkers (|cor| > 0.3, *p* < 0.05).

### 2.8 Construction of regulatory network

So as to investigate the possible expression regulation patterns of the potential biomarkers, the miRWalk database was applied to predict miRNAs targeting them. Subsequently, to identify the transcription factors (TFs) interacting with potential biomarkers, the miRNet database was applied to predict the relevant TFs. Moreover, we employed the CTD method to screen for drug candidates targeting potential biomarkers of KD.

### 2.9 Reverse transcription quantitative PCR (RT-qPCR)

The expression profiles of the potential biomarkers in clinical samples were determined by RT-qPCR. This study was approved by the Ethics Committee of Fujian Children's Hospital (approval number: 2026ETKRLK02006, valid period: February 9, 2026 - February 9, 2027), and written informed consent was obtained from the guardians of all participants.

KD patients were enrolled based on the 2017 American Heart Association (AHA) diagnostic guidelines for acute-phase KD (30). Inclusion criteria for KD group: aged ≤10 years; fever duration ≤10 days before blood collection; no prior intravenous immunoglobulin (IVIG) or glucocorticoid treatment. Exclusion criteria for KD group: incomplete KD; combined with other autoimmune diseases, infectious diseases or malignant tumors; history of KD. Inclusion criteria for healthy control group: healthy children undergoing physical examination in the Physical Examination Center of Fujian Children's Hospital during the same period; no history of KD, autoimmune diseases, acute infections or chronic inflammatory diseases; frequency-matched with KD group in age and sex. From February 2026 to April 2026, 5 acute-phase KD patients and 5 healthy controls were consecutively recruited from the Department of Cardiovascular Medicine and Physical Examination Center of Fujian Children's Hospital, respectively. Peripheral venous blood (2 mL) was collected into EDTA anticoagulant tubes, and blood cells were isolated within 4 h and stored at −80℃ with TRIzol reagent (Invitrogen, USA) for subsequent RNA extraction.

Clinical data of KD patients, including age, sex, fever duration before blood collection, coronary artery lesions (CALs) status and IVIG responsiveness, were collected from electronic medical records. IVIG non-response was defined as persistent fever ≥38℃ at 36 h after IVIG treatment. The clinical characteristics of all subjects are detailed in Table 1.

**Table 1:** Clinical and demographic characteristics of the RT-qPCR validation cohort.

Characteristic		KD1	KD2	KD3	KD4	KD5	HC1	HC2	HC3	HC4	HC5
Demographics	Sex (Male/Female)	M	M	M	F	F	M	F	M	M	F
	Age (years)	4.4	1	1.5	1.25	3.3	4	2.25	1.3	2.75	1.2
Disease characteristics	KD type	Incomplete	Complete	Complete	Complete	Complete	-	-	-	-	-
	Fever days at blood draw (days)	7	6	6	6	5	-	-	-	-	-
	Pre-blood draw IVIG treatment	No	No	No	No	No	-	-	-	-	-
IVIG treatment response (post-blood draw)	IVIG response	Sensitive	Sensitive	Sensitive	Sensitive	Resistant	-	-	-	-	-
Coronary artery lesion (CAL)	CAL status	Small aneurysm	Normal	Normal	Normal	Smal aneurysm	-	-	-	-	-
	Max Z-score	3.01	1.77	0.05	1.5	3.57	-	-	-	-	-
Treatment (post-blood draw)	Treatment received	IVIG	IVIG	IVIG	IVIG	IVIG + steroid	-	-	-	-	-

All blood samples from KD patients were collected before IVIG administration. IVIG was given as standard clinical treatment after blood draw. KD5 was IVIG-non-responsive and subsequently received additional glucocorticoid therapy.

Coronary artery lesion (CAL) criteria (Z-score):No involvement: Always <2,Dilation only: 2 to <2.5; or if initially <2, a decrease in Z score during follow-up ≥1,Small aneurysm: ≥2.5 to <5,Medium aneurysm: ≥5 to <10, and absolute dimension <8 mm,Large or giant aneurysm: ≥10, or absolute dimension ≥8 mm.According to these criteria, KD1 (Z = 3.01) and KD5 (Z = 3.57) had small aneurysms, KD, kawasaki disease; HC, healthy control; IVIG, intravenous immunoglobulin; CAL, coronary artery lesion.

The total RNA of the samples was extracted by the Trizol method (Vazyme, R401-01, China) (31), and the cDNA was synthesized via the HP All-in-one qRT Master Mix II RT203-Ver.1 (Younggenbio, 24Y0124, China). Finally, RT-qPCR was performed with the ® 2 × Universal Blue SYBR Green qPCR Master Mix kit (Servicebio, G3326-05, China). The 2-*ΔΔ*Ct method was employed to compute potential biomarker expression. Primer sequences are shown in Supplementary Table S2.

### 2.10 Statistical analysis

The R (v 4.2.2) was used to execute bioinformatics analysis. The Wilcoxon test was adopted to evaluate paired sample comparisons, and the difference between two groups in the RT-qPCR was determined using the t-test (*p* < 0.05).

## 3 Results

### 3.1 The relevance of candidate genes to MD was revealed

**Figure 1:**
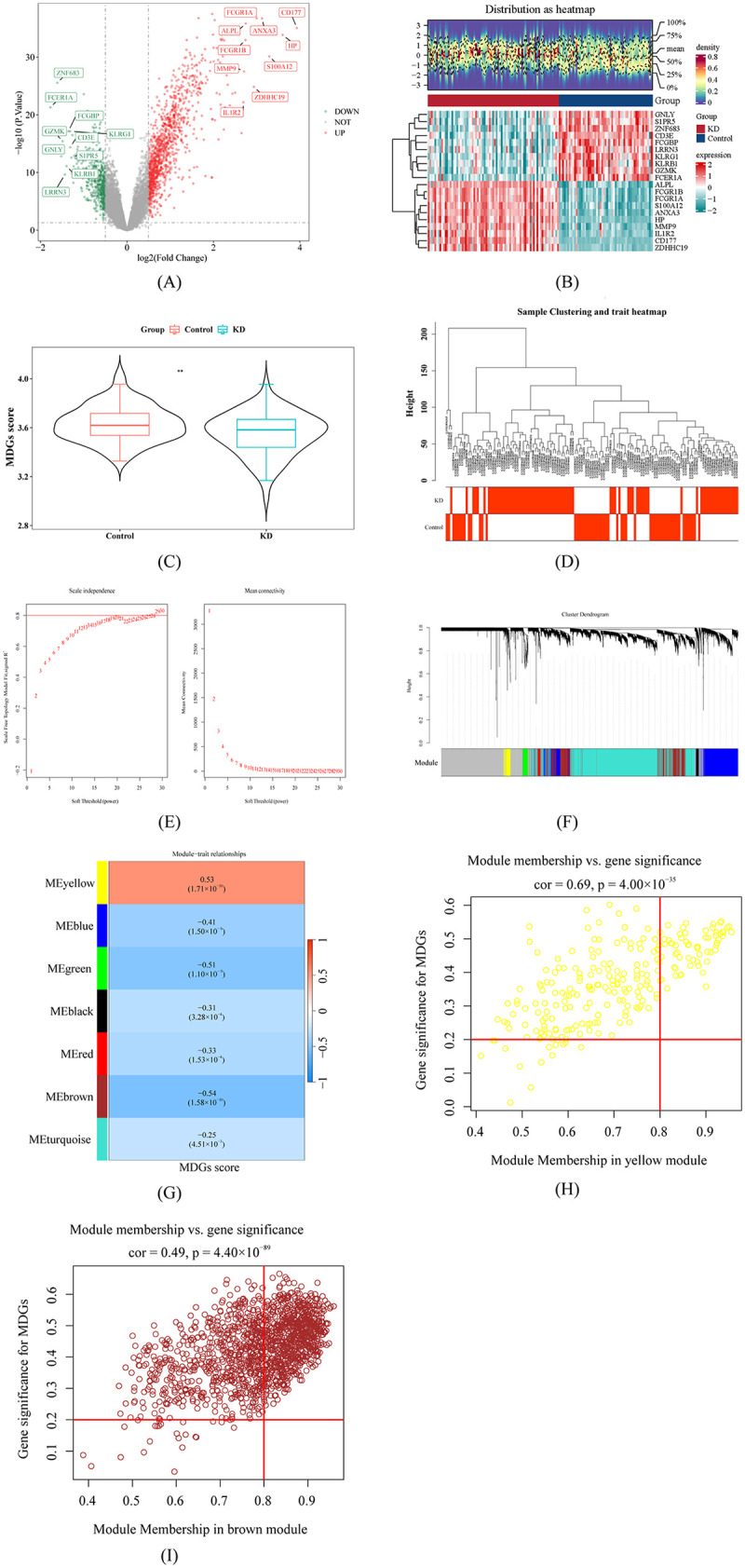
Identification of differentially expressed genes and WGCNA analysis. **(A)** Volcano plot of DEGs between KD and control groups. **(B)** Heatmap of top 20 DEGs. **(C)** MDGs scores comparison between groups. **(D)** Sample clustering dendrogram. **(E)** Scale-free fit index for soft threshold selection. **(F)** Gene dendrogram with co-expression modules. **(G)** Module-trait correlation heatmap. **(H,I)** Module membership vs. gene significance scatter plots for yellow and brown modules.

Following strict QC and independent preprocessing of GSE73461, no outlier samples were excluded, and gene expression data showed good normalization and homogeneity. After screening, the 1,688 DEGs were identified, among which 1,152 were up-regulated DEGs, and 536 were down-regulated DEGs in the KD samples, and DEGs between groups exhibited distinct profiles (Figures 1A,B). Batch effect assessment via PCA confirmed no significant technical heterogeneity within GSE73461, and the independent validation set GSE68004 showed consistent expression distribution with the training set, validating the reliability of the batch effect control strategy. Subsequently, MDG scores in the KD group were significantly lower than in the control group (*p* < 0.01) (Figure 1C). Following exclusion of no eligible samples, all GSE73461 samples were used for WGCNA analysis (Figure 1D). Then, the optimal soft threshold (power) was 29 (R^2^ = 0.80) (Figure 1E), and 7 modules were identified (excluding gray modules that cannot be categorized) (Figure 1F). The yellow module and the brown module were selected (Figure 1G). Following this, 835 key module genes were identified, among which 62 and 773 genes were acquired from the yellow and brown modules, respectively (Figures 1H,I).

**Figure 2:**
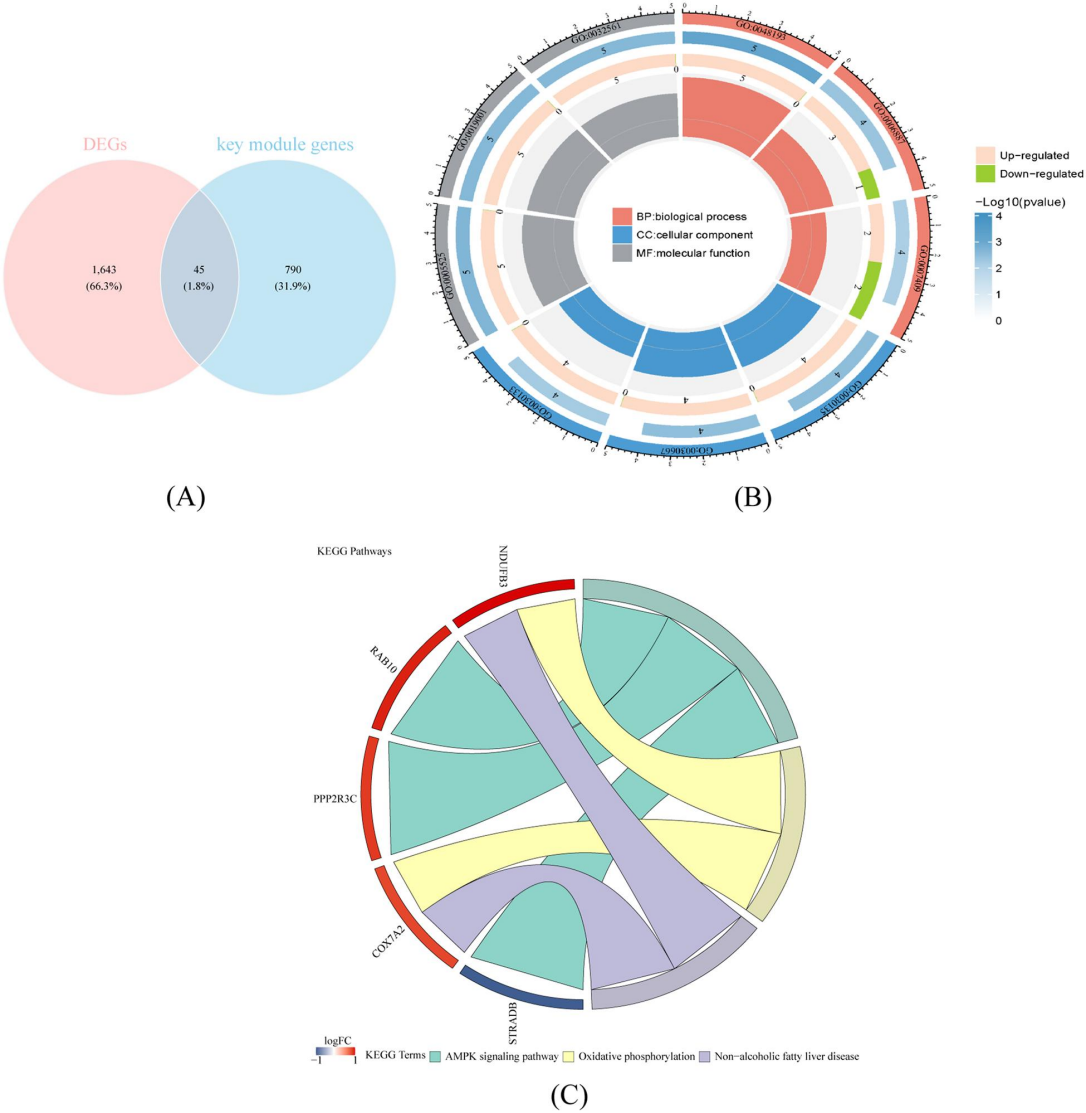
Functional enrichment analysis of candidate genes. **(A)** Venn diagram showing candidate genes obtained from DEGs and MDGs. **(B)** Gene Ontology (GO) analysis of the candidate genes. **(C)** KEGG enrichment analysis of the candidate genes. DEGs, differentially expressed genes; MDGs, mucosal disease genes.

Eventually, 45 candidate genes were retained (Figure 2A). Moreover, the candidate genes were markedly enriched in 197 GO entries (Supplementary Table S3). Specifically, there was a marked enrichment mainly in axonogenesis (BP), transport vesicle (CC) and GTP binding (MF) (Figure 2B). Meanwhile, candidate genes were markedly enriched in 3 KEGG pathways, including AMPK signaling pathway, oxidative phosphorylation and non-alcoholic fatty liver disease (Figure 2C). These enriched pathways were closely associated with mitochondrial energy metabolism, endothelial cell homeostasis, and inflammatory regulation, suggesting that the candidate genes might be involved in coronary artery lesion progression in KD by modulating energy metabolism and inflammatory responses. Notably, several of the 23 MDGs (e.g., MFN1, MFN2, OPA1, and DNM1L/DRP1) constitute the canonical mitochondrial fission/fusion machinery. The identification of candidate genes from these MDGs indicates that the genes may also influence the balance of mitochondrial fission and fusion, beyond regulating energy metabolism and inflammation.

### 3.2 RAP2C, DPM2, and DDX59 were MD-related potential biomarkers in KD

**Figure 3:**
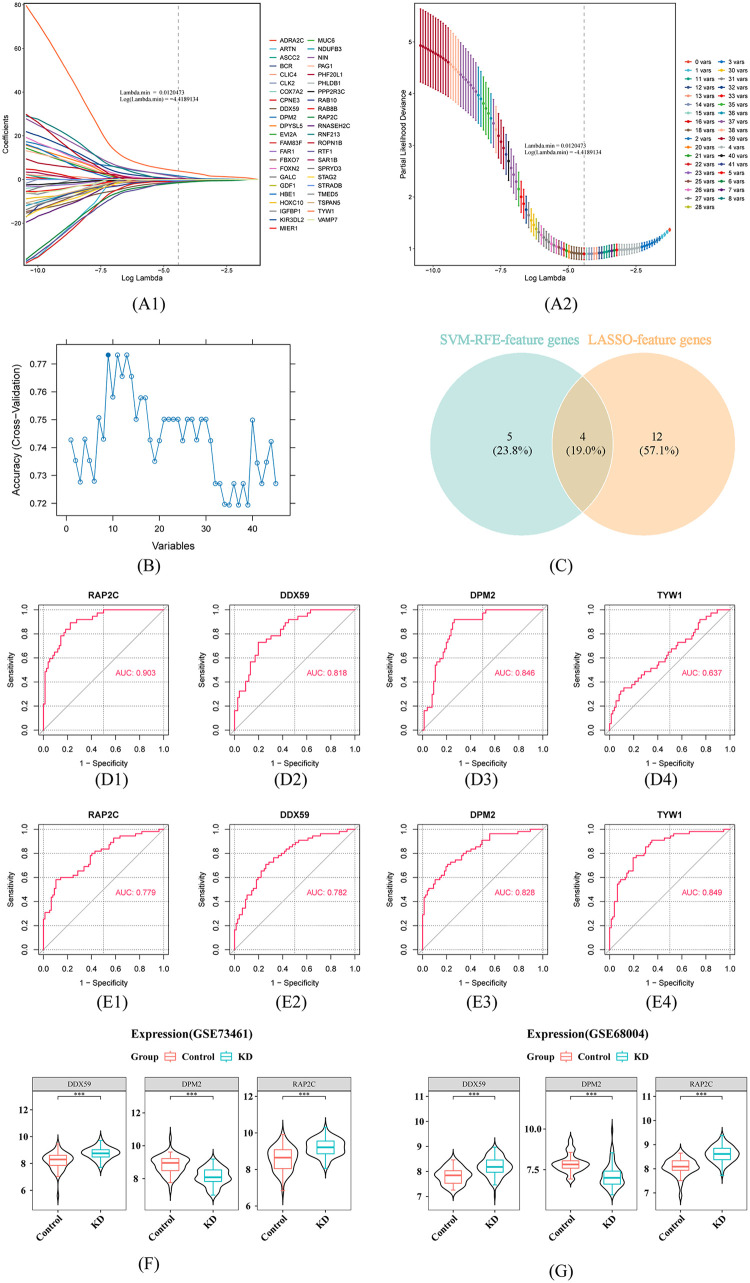
Screening of Key genes. (**A1**) LASSO coefficient profile plot. (**A2**) Ten-fold cross-validation for tuning parameter selection in the LASSO analysis. **(B)** Candidate gene screening by SVM-RFE analysis. **(C)** Venn diagram showing the intersection of feature genes identified by the two algorithms. ROC curves in **(D)** GSE73461 and **(E)** GSE68004 datasets. Expression levels of potential biomarkers in **(F)** GSE73461 and **(G)** GSE68004 dataset.

The 16 LASSO-feature genes were acquired after LASSO regression analysis [lambda.min = 0.0120473, log(lambda.min) = −4.4189134] (Figure 3A), including ASCC2, STRADB, CPNE3, NIN, ADRA2C, EVI2A, GDF1, RAP2C, RTF1, TYW1, DPM2, DDX59, KIR3DL2, SAR1B, CLIC4, and HOXC10. Meanwhile, 9 SVM-RFE-feature genes were acquired when the SVM-RFE model reached the highest accuracy, including TYW1, DPM2, DDX59, RAP2C, RAB10, PHLDB1, FAR1, ROPN1B, and FOXN2 (Figure 3B). Afterwards, 4 feature genes were acquired by intersection, namely RAP2C, TYW1, DPM2 and DDX59 (Figure 3C). However, TYW1 was excluded because its AUC value in the validation set GSE68004 was less than 0.7, failing to meet the pre-set screening threshold for diagnostic efficacy. In both the GSE73461 and GSE68004, RAP2C (AUC > 0.7), DPM2 (AUC > 0.8), and DDX59 (AUC > 0.7) were shown to have the ability to discriminate well between KD and control samples, thus serving as potential biomarkers (Figures 3D,E). Notably, the RAP2C and DDX59 were markedly up-regulated, and conversely, DPM2 was markedly down-regulated in the KD group (*p* < 0.0001) (Figures 3F,G).

### 3.3 Nomogram had a favorable performance in predicting KD

**Figure 4:**
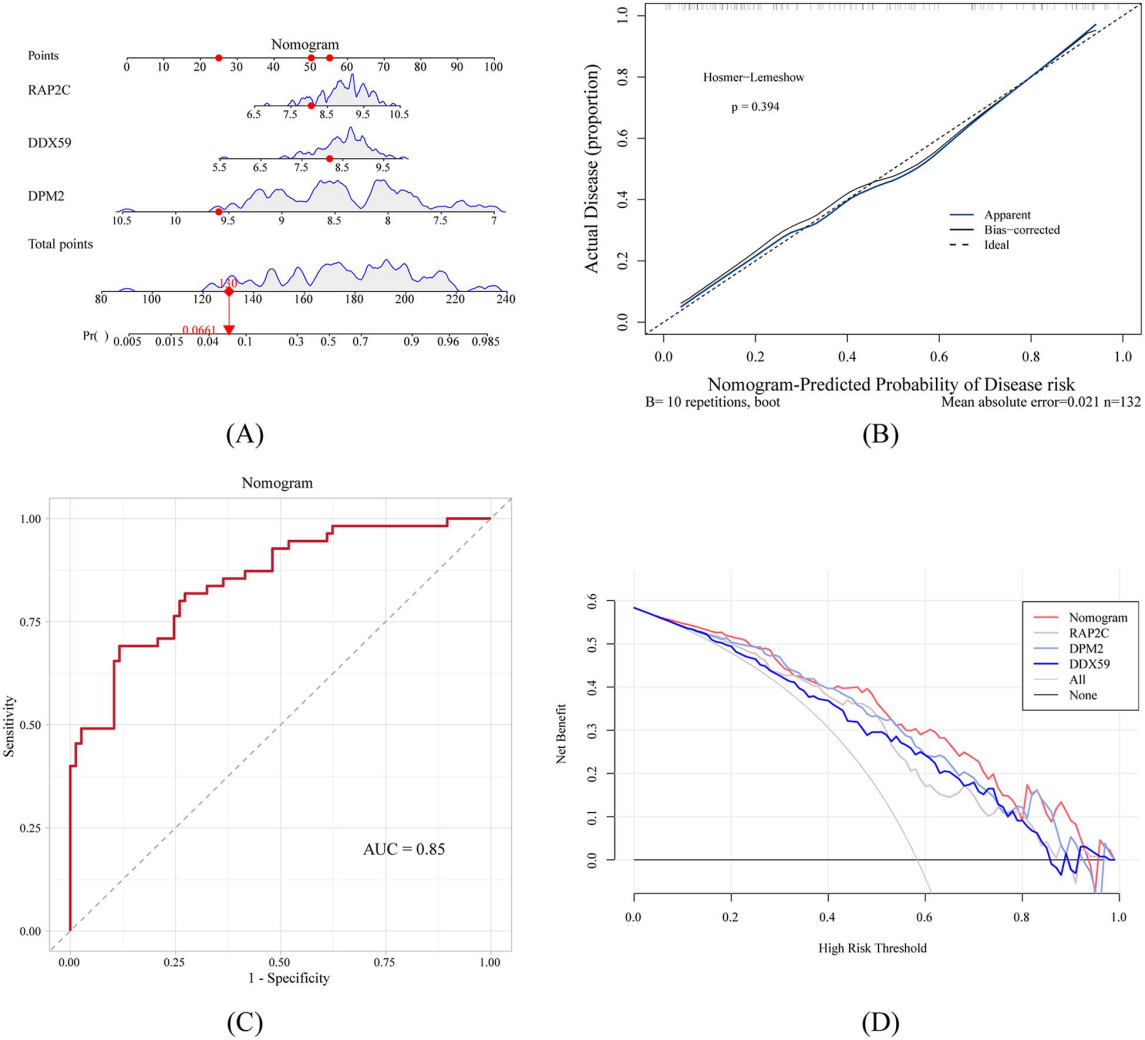
Construction of the nomogram model. (**A**) Nomogram for predicting the occurrence of KD. (**B**) Calibration curve of the nomogram model. (**C**) ROC curve of the nomogram model. (**D**) DCA of the nomogram model.

Based on RAP2C, DPM2, and DDX59, predicting incidence of KD was displayed in the nomogram, with the higher the total points, the higher the probability of KD (Figure 4A). Subsequently, the calibration curve showed no significant deviation between predicted and actual values (Hosmer-Lemeshow test, *p* = 0.394), with an AUC of 0.85, indicating good predictive accuracy of the nomogram (Figures 4B,C). In addition, DCA revealed that the nomogram achieved a positive net benefit, which was higher than that of the all-or-none strategy and individual potential biomarkers (Figure 4D). Collectively, the nomogram established using these potential biomarkers exhibited satisfactory predictive performance for KD risk, providing a valuable clinical decision-making tool.

### 3.4 Targeting these key pathways might offer novel ideas for KD treatment

**Figure 5:**
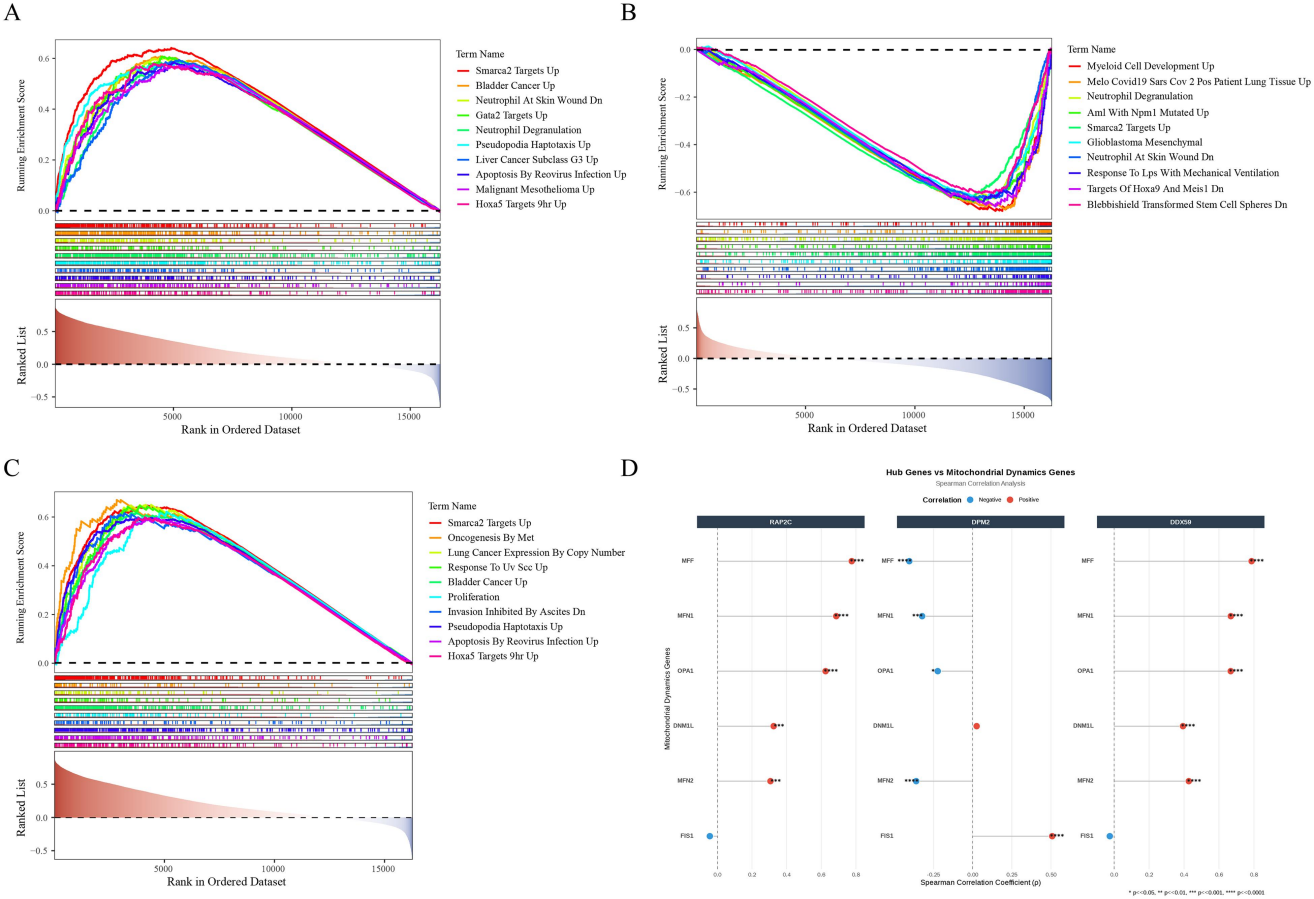
Gene Set enrichment analysis (GSEA). **(A)** GSEA pathway enrichment analysis for the potential biomarker DDX59. **(B)** GSEA pathway enrichment analysis for the potential biomarker DPM2. **(C)** GSEA pathway enrichment analysis for the potential biomarker gene RAP2C. **(D)** Lollipop plots of Spearman correlation analyses between the three potential biomarkers and core mitochondrial dynamics genes.

Afterwards, bioinformatic prediction via GSEA was performed to further understand how potential biomarkers might be involved in KD development. The results displayed that DDX59 was markedly enriched in neutrophil at skin wound DN, neutrophil degranulation, and apoptosis by reovirus infection UP (Figure 5A). DPM2 was markedly enriched in myeloid cell development UP, neutrophil degranulation, and neutrophil at skin wound DN (Figure 5B). RAP2C was markedly enriched in SMARCA2 targets UP, proliferation, and apoptosis by reovirus infection UP (*p* < 0.05, |NES| > 1) (Figure 5C). Overall, these key pathways revealed the relevance of potential biomarkers to inflammation-related pathways and also illustrate the relevance of potential biomarkers to KD pathology. Spearman's correlation analysis further revealed that the three potential biomarkers exhibited distinct patterns of association with core MD genes (Figure 5D). Both RAP2C and DDX59 showed significant positive correlations with DNM1L, MFN1, MFN2, OPA1, and MFF, with the strongest correlation observed for MFF (*p* < 0.05). In contrast, DPM2 showed a significant positive correlation with FIS1, while exhibiting significant negative correlations with all four core genes (MFN1, MFN2, OPA1, and MFF), with the strongest correlation observed for FIS1 (*p* < 0.05).

### 3.5 The immune microenvironment of KD patients was altered

**Figure 6:**
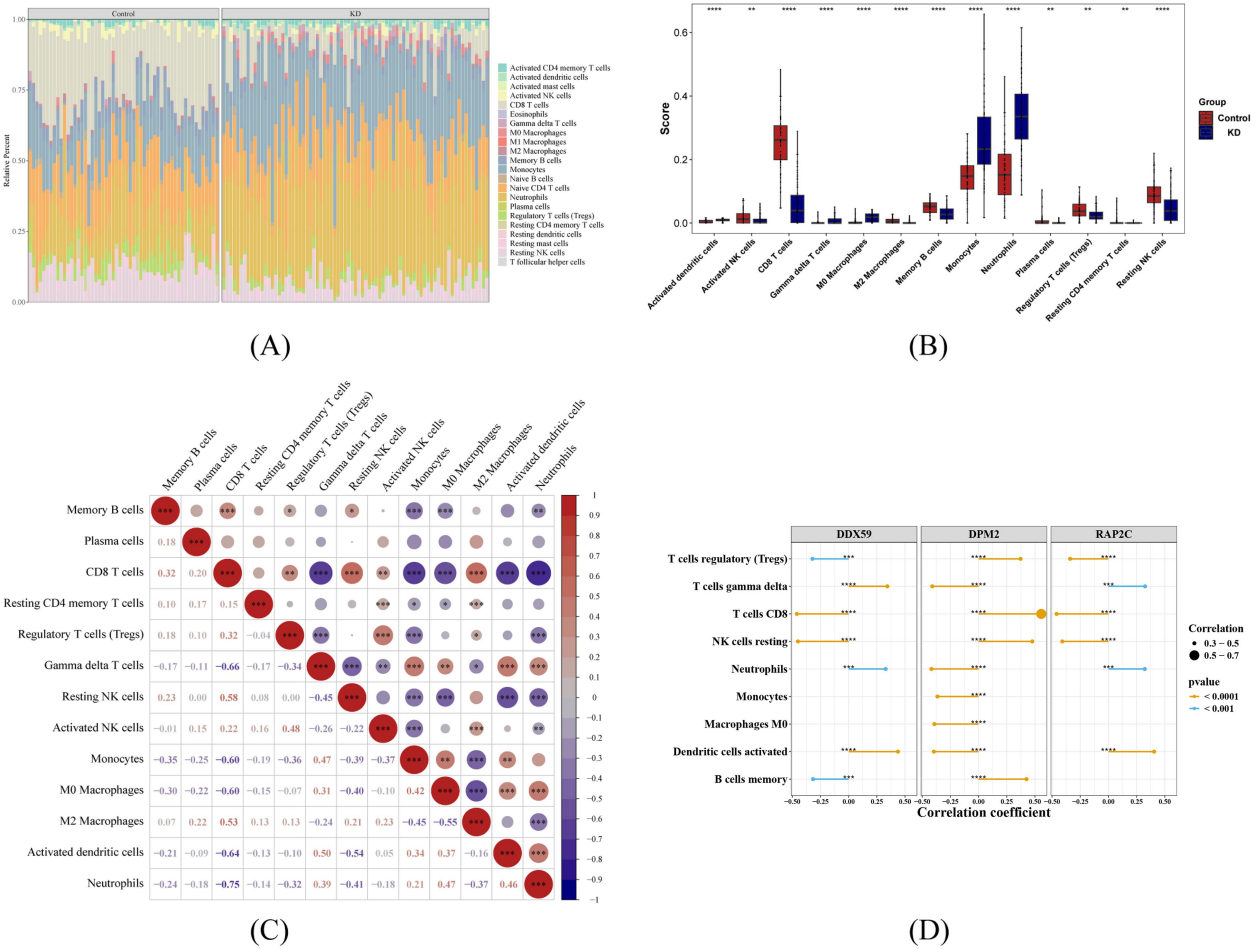
Immune cell infiltration analysis and immune correlation analysis. **(A)** Stacked bar chart showing the proportions of immune cell infiltration in KD and control samples. **(B)** Comparison of the proportions of 13 immune cell types between the KD and control groups, presented as box plots. **(C)** Correlation matrix of differential immune cells and their statistical significance. Red indicates a positive correlation, with darker red representing a stronger positive correlation; blue indicates a negative correlation, with darker blue representing a stronger negative correlation. **(D)** Correlation between potential biomarkers and differential immune cells. The *x*-axis represents potential biomarkers, and the *y*-axis represents differential immune cells. Positive values indicate a positive correlation, negative values indicate a negative correlation, and zero indicates no correlation. *Asterisks denote statistical significance: *p < 0.05, **p < 0.01, ***p < 0.001, **p < 0.0001. KD, Kawasaki disease.

According to the KD and control groups, the immune cell infiltration was shown in Figure 6A, which displayed a higher relative abundance of monocytes, neutrophils in the KD group and follicular helper T cells in the control group. Next, 13 immune cells were markedly different between groups (*p* < 0.05). Among them, 6 differential immune cells had higher ssGSEA scores in the KD group, namely activated dendritic cells, gamma delta T cells, M0 macrophages, monocytes, neutrophils, and regulatory T cells (Tregs) (Figure 6B). Then, the relationships were observed between differential immune cells, such as resting natural killer (NK) cells had the strongest positive relevance with CD8 T cells, and the strongest negative relevance was observed between neutrophils and CD8 T cells (*p* < 0.001) (Figure 6C). Notably, there were marked relationships between the potential biomarkers and 9 differential immune cells. Specifically, DPM2 exhibited the strongest positive correlation with CD8 T cells (r = 0.72,*p* = 2.1 × 10^−5^), while DDX59 showed the strongest negative correlation with CD8 T cells (r = −0.68,*p* = 5.3 × 10^−6^) (Figure 6D, Supplementary Table S4). The disordered immune cell infiltration and strong correlations between potential biomarkers and immune cells further indicate that immune microenvironment imbalance is a key factor associated with vascular inflammation and coronary artery lesions in KD.

### 3.6 Regulatory networks provided the basis for targeted regulation of potential biomarkers

**Figure 7:**
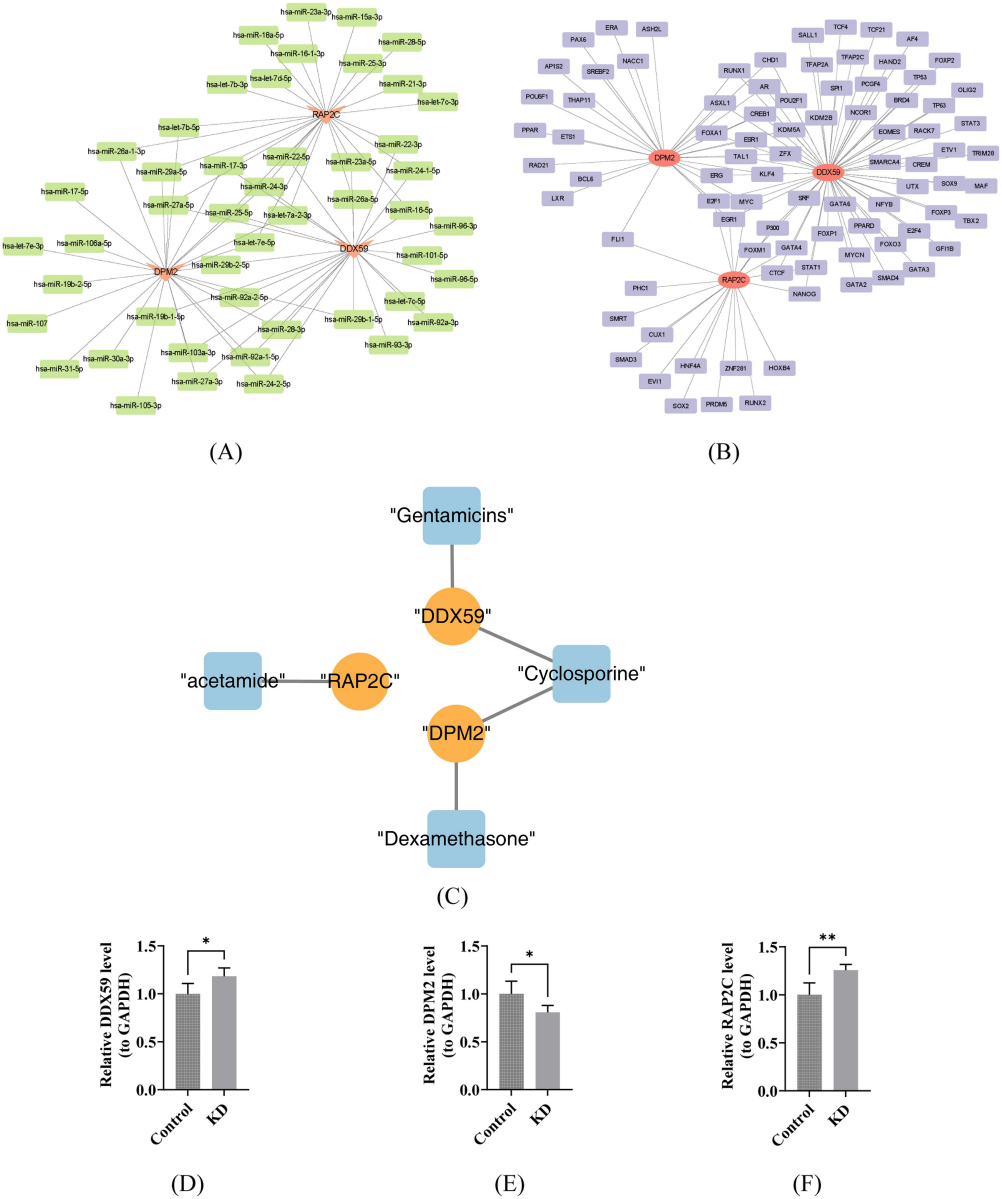
Regulatory networks. **(A)** miRNA-potential biomarker (mRNA) regulatory network. Potential biomarkers (mRNAs) are shown in orange, and miRNAs are shown in green. **(B)** Potential biomarker-TFs regulatory network. Potential biomarkers are shown in red, and transcription factors predicted from the potential biomarkers are shown in purple. **(C)** Substance-potential biomarker interaction network. Yellow nodes represent the three potential biomarkers (RAP2C, DPM2, DDX59); blue nodes represent the four low-toxicity candidate substances (acetamide, Gentamicins, Dexamethasone, Cyclosporine) screened by ADMETlab 3.0 after excluding toxic substances. **(D–F)** RT-qPCR validation of RAP2C, DDX59, and DPM2 expression in KD vs. control samples. *p < 0.05.

The 5,016, 1,561, and 3,443 miRNAs targeting RAP2C, DPM2, and DDX59 were predicted, respectively. There were 48 miRNAs for each potential biomarker score top 30 after removal of duplicates. Among them, RAP2C, DPM2, and DDX59 shared 5 common miRNAs, namely hsa-miR-17-3p, hsa-miR-24-3p, hsa-miR-25-5p, and hsa-miR-27a-5p, revealed potential co-expression correlations among potential biomarkers (Figure 7A). Subsequently, the 21, 29, and 62 TFs targeting RAP2C, DPM2, and DDX59 were predicted, respectively. Notably, a total of 3 TFs could co-regulate potential biomarkers, including E2F1, MYC, and EGR1. This convergence on common upstream regulators indicates that these functionally distinct genes are involved in a coordinated biological response in the context of KD. And the regulatory network of DDX59 was relatively complex compared to DPM2 and RAP2C (Figure 7B). Overall, the regulatory network supplied a theoretical basis for targeting and modulating potential biomarkers for the treatment of KD.

Moreover, the construction of drug regulatory networks further revealed the clinical potential of targeting potential biomarkers to treat KD. Notably, the Comparative Toxicogenomics Database (CTD)records both toxicological and therapeutic interactions between chemical substances and genes, rather than only containing therapeutic target information. Specifically, RAP2C, DPM2, and DDX59 predicted 53, 40, and 57 interacting chemical substances, respectively. Among the top 30 substances ranked by interaction count for each potential biomarker, common substances were preliminarily identified. It should be emphasized that benzo(a)pyrene, a common preliminary prediction result, is classified as a Group 1 carcinogen by the International Agency for Research on Cancer (IARC) and is an environmental pollutant with no therapeutic value, so it was excluded from subsequent screening.

To obtain clinically plausible candidates, we further used ADMETlab 3.0(https://admetlab3.scbdd.com/server/screening) to evaluate the toxicity of 70 initially screened substances. The screening focused on five key toxicity parameters: hERG blockade, human drug-induced liver injury (DILI), eye corrosion, respiratory toxicity, and oral acute toxicity in rats, with a risk probability ≥0.5 defined as high risk. After strict filtering, four low-toxicity substances (acetamide, Gentamicins, Dexamethasone, Cyclosporine) were identified as candidate substances co-targeted DDX59, RAP2C, and DPM2 (Figure 7C, Table 2). In short, these predicted drug-gene interactions provide clues for future drug repurposing studies of KD, rather than indicating definite therapeutic effects.

**Table 2:** Low-toxicity candidate substances screened by ADMETlab 3.0.

drug	hERG	DILI	EC	Respiratory	LD50_oral
acetamide	0.0821199119091034	0.28758841753006	0.34430056810379	0.401542186737061	[‘-’]
Gentamicins	0.44730931520462	0.000400799210183322	2.8335884394437e-07	0.306144177913666	[‘-’]
Dexamethasone	0.0736724585294724	0.01241594273597	2.44381444645114e-05	0.471715420484543	[‘-’]
Cyclosporine	0.00110287114512175	0.218412384390831	9.30994929823344e-21	3.7317760870792e-05	

### 3.7 Preliminary validation of potential biomarker expression in a small clinical cohort

RT-qPCR analysis revealed that RAP2C and DDX59 were significantly up-regulated, whereas DPM2 was markedly down-regulated in KD samples (*p* < 0.05) (Figures 7D–F). These expression patterns were consistent with the trends observed in the GSE73461 and GSE68004 datasets, providing preliminary support for the reliability of the potential biomarker screening; however, their diagnostic value in distinguishing KD from control samples requires further validation in larger cohorts.

## 4 Discussion

As a primary cause of acquired heart disease in children, KD represents a self-limiting, multisystemic vasculitis that predominantly impacts the coronary arteries.This study is a pure bioinformatics analysis, and the conclusions are only predictions at the bioinformatics level. It has not been verified by *in vitro* cell experiments or *in vivo* animal experiments, and cannot confirm the direct regulatory effects of RAP2C, DPM2, and DDX59 on mitochondrial dynamics and CD8+ T cell infiltration. Its heterogeneity contributes to diagnostic challenges, making precise biomarkers crucial (32). Recent studies indicate that mitochondrial dysfunction participates in KD through mechanisms such as mitochondrial DNA release and excessive ROS production (14). However, a systematic investigation into the role of MD-related genes in KD remains lacking. Therefore, this study employed transcriptomics and validate three MD-related potential biomarkers for KD: RAP2C, DPM2, and DDX59. These potential biomarkers exhibited favorable diagnostic efficacy in both the training dataset and an independent validation dataset. Nomogram and ROC curve analyses further confirmed their potential application value in the diagnosis of KD, providing a novel theoretical basis for early identification and intervention of the disease. GSEA revealed that these three potential biomarkers were collectively enriched in inflammation-related pathways, including neutrophil degranulation and apoptotic regulation, suggesting their potential involvement in KD pathogenesis via the modulation of inflammatory responses. Immune infiltration analysis identified a significant increase in monocytes and neutrophils in KD patients, and the potential biomarkers showed significant correlations with immune cell infiltration levels, indicating that they may influence disease progression by altering the immune microenvironment. Furthermore, regulatory network analysis untangled five common miRNAs and three common transcription factors (E2F1, MYC, EGR1) that could be associated with these potential biomarkers, and identified three potential targeted drugs. These results offer novel theoretical insights and therapeutic targets for the early diagnosis and precise therapy of KD. Taken together, these bioinformatic findings suggest a potential mitochondrial dysfunction-inflammation-immune imbalance axis associated with these three biomarkers, which may be relevant to coronary artery injury in KD. It should be emphasized that this proposed mechanism is a hypothesis derived from correlation analysis; further mechanistic studies and preclinical investigations are needed to evaluate its therapeutic significance.

Notably, although RAP2C, DPM2, and DDX59 are predicted to have distinct molecular functions—potentially regulating mitochondrial fission/fusion, mediating protein glycosylation, and modulating RNA metabolism, respectively—our bioinformatics analysis suggests they may converge on the core theme of mitochondrial dynamics through shared upstream regulatory networks and synergistic effects on mitochondrial homeostasis. First, as revealed in our regulatory network analysis, the three genes are predicted to be co-regulated by 5 common miRNAs (e.g., hsa-miR-17-3p) and 3 common transcription factors (E2F1, MYC, EGR1). This shared upstream regulation, identified computationally, suggests they might be components of a coordinated biological program responding to cellular stress in KD. Second, their functional impacts intersect at the level of mitochondrial protein integrity and function: RAP2C directly modulates the core fission machinery (DRP1) to maintain fusion-fission balance; DPM2-mediated glycosylation is critical for the stability and correct folding of outer mitochondrial membrane proteins (e.g., mitofusins); DDX59 ensures efficient translation of mitochondrial dynamics-related proteins via regulating ribosome biogenesis. Dysregulation of any of these potential biomarkers disrupts the delicate balance of mitochondrial dynamics, ultimately contributing to mitochondrial dysfunction and vascular inflammation in KD. However, all of these functional relationships are derived from literature or bioinformatics predictions in the context of other diseases, and their relevance to KD requires experimental validation.

RAP2C, a member of the Rap superfamily discovered by Paganini et al., functions as a molecular switch in various signal transduction pathways and is primarily involved in cell/matrix adhesion, cell/cell adhesion, and cytoskeletal rearrangement (33). RAP2C is predominantly localized in the cytoplasm and plasma membrane, and its silencing typically leads to disruption of cellular junctions. As a GTPase, RAP2C interacts with the mitochondrial fission regulator protein DRP1 to promote phosphorylation at the S637 site of DRP1, thereby facilitating mitochondrial fusion and oxidative phosphorylation. Conversely, silencing of RAP2C results in increased mitochondrial fission, elevated production of ROS, and cytochrome C release (34). Studies have demonstrated that RAP2C associates with vascular permeability and barrier function in endothelial cells, contributing to the maintenance of endothelial homeostasis (35). In the pathophysiology of KD, endothelial cells, located on the luminal surface of coronary arteries, serve as a critical interface between circulating inflammatory cells and the vascular wall and are the primary target of the early inflammatory assault in KD. Significant vascular endothelial cell injury and dysfunction are observed in KD patients. Endothelial dysfunction is recognized as a key contributor to KD pathogenesis and serves a critical role in the development of coronary artery aneurysms (36). Based on literature evidence from other disease contexts, it is hypothesized that RAP2C may influence endothelial barrier function through its reported effects on mitochondrial function and oxidative stress. Our bioinformatic analysis revealed that RAP2C expression is altered in KD and shows correlation with mitochondrial dynamics genes. Combining these computational observations with literature evidence, we propose that abnormal expression of RAP2C may be correlated with vascular endothelial injury in KD, potentially through effects on mitochondrial homeostasis and oxidative stress. However, this hypothesis is derived from bioinformatic predictions and literature extrapolation, and requires experimental validation in KD-specific models.

Beyond its role in endothelial function, RAP2C interacts directly with the core machinery governing mitochondrial fission. As a GTPase, RAP2C has been shown to interact with dynamin-related protein 1 (DRP1), a master regulator of mitochondrial fission (34). Specifically, RAP2C promotes DRP1 phosphorylation at the S637 site, which suppresses excessive mitochondrial fission and favors mitochondrial fusion and oxidative phosphorylation (34). For DPM2, its downregulation in KD may indirectly affect the stability of mitofusins (MFN1/2), key mediators of mitochondrial fusion, by impairing mitochondrial protein quality control and lipid remodeling. As for DDX59, although it is traditionally linked to RNA helicase activity, its involvement in mitochondrial ribosome biogenesis, by extension, raises the possibility of a correlation with the translation of core fission/fusion proteins. We speculate that dysregulation of these three potential biomarkers might be associated with an imbalance between MFN1/2-mediated fusion and DRP1-mediated fission, which could potentially be related to mitochondrial fragmentation observed in KD vasculitis.

DPM2 encodes a mannosyl-phospho-dolichol utilization defect type glycosyltransferase and is associated with congenital disorders of glycosylation (CDG). Dysregulation of DPM2 can lead to defects in the glycosylation of proteins and lipids. Its deficiency affects protein expression and glycosylation processes and is linked to iron metabolism abnormalities resulting from defects in mitochondrial fatty acid synthesis (37). Intravenous immunoglobulin (IVIG) is recommended as the first-line therapeutic agent for KD. Studies have shown that following IVIG treatment, plasma hepcidin levels in patients with KD significantly decrease, accompanied by changes in hemoglobin levels. The alteration in hepcidin levels is closely associated with IVIG treatment resistance and the formation of coronary artery lesions (38). Furthermore, studies have investigated the utility of GlycA, a protein glycosylation signature, in differentiating acute KD from other febrile conditions. Analyses demonstrated that elevated GlycA levels in KD patients substantiate that increased protein glycosylation constitutes a prominent feature of the acute-phase response (39). Based on literature evidence linking DPM2 to glycosylation disorders and iron metabolism, we speculate that DPM2 might be associated with the acute-phase inflammatory response in KD, possibly through its known role in protein glycosylation. Our bioinformatic analysis revealed dysregulated DPM2 expression in KD, and its correlation with immune cell infiltration suggests a potential association with disease severity and treatment response. However, these findings are purely computational, and the hypothesized relationship between DPM2 and IVIG responsiveness or coronary artery injury risk requires prospective clinical validation and mechanistic studies.

DDX59 belongs to the DEAD-box RNA helicase family and participates in diverse RNA metabolic processes, such as RNA splicing, nuclear export, and ribosome assembly. Some members of this family exhibit subcellular localization associated with mitochondrial RNA metabolism (40). The ribosome, as the central organelle for protein synthesis in cells, can lead to multifaceted pathological effects when its metabolism is disordered, including abnormalities in ribosome assembly and protein translation efficiency. First, aberrant synthesis of immune-related proteins, such as cytokines and immune receptors, may disrupt the balance of the immune response in patients with KD, thereby exacerbating vascular inflammation. Systemic vasculitis is a core pathological feature of KD. Second, impaired synthesis of structural or functional proteins required by vascular endothelial cells can compromise vascular endothelial integrity, consequently increasing the risk of severe complications such as coronary artery aneurysms (41). Based on literature reports linking DDX59 to RNA metabolism and ribosome biogenesis, we speculate that DDX59 might be associated with immune dysregulation and vascular inflammation in KD, potentially via its predicted involvement in protein synthesis in immune and endothelial cells. Our computational analysis revealed correlations between DDX59 expression and immune cell infiltration, supporting a potential link. However, this hypothesis is based solely on bioinformatic predictions and literature extrapolation, and requires direct experimental testing in KD-relevant cell and animal models.

The GSEA enrichment analysis in this study revealed that both DDX59 and DPM2 were enriched in the neutrophil degranulation pathway, which is a pathway previously implicated in the pathophysiology of KD. Previous studies have confirmed a significant expansion of CD177 + neutrophils in the blood of KD patients. These cells exhibit highly activated effector functions, with the neutrophil degranulation pathway being specifically activated. This pathway is associated with the SPI1 gene, and its excessive activation may lead to systemic pathological damage and be involved in the development of coronary artery complications (42). Mechanistically, neutrophils participate in tissue injury through multiple pathways, including degranulation-mediated release of toxic mediators, ROS generation via NADPH oxidase triggering oxidative stress injury, and formation of neutrophil extracellular traps (NETs). This respiratory burst phenomenon and excessive NET formation are closely associated with the risk of coronary artery lesions in KD, participating in the pathological process of KD vasculitis by inducing vascular endothelial injury and amplifying inflammatory effects (43, 44). In summary, abnormal activation of the neutrophil degranulation pathway is one of the important pathological mechanisms underlying vascular inflammation and coronary artery injury in KD. The enrichment of the potential biomarkers DDX59 and DPM2 identified in this study within this pathway provides preliminary bioinformatic evidence for their potential involvement in the pathogenesis of KD, warranting further investigation.

Immunoinfiltration analysis in this study revealed a significant positive correlation between resting NK cells and CD8+ T cells, whereas neutrophils showed a significant negative correlation with CD8+ T cells, untangling the complex network of immune cell interactions in KD. Further analysis demonstrated significant associations between the potential biomarkers and nine types of differential immune cells. Notably, DPM2 exhibited the positive correlation with CD8+ T cells, while DDX59 showed the strongest negative correlation, suggesting that these biomarkers might be associated with the immunopathological process of KD, potentially via their correlation with CD8+ T cell function. Extensive research has confirmed the central role of CD8+ T cells in the development of coronary arteritis in KD: in mouse models, CD8+ T cells (but not CD4+ T cells) are essential for the occurrence of coronary artery lesions and functionally promote the progression of KD vasculitis (45). Pathological studies further indicate that the predominant lymphocyte subset infiltrating coronary artery lesion tissues during the acute phase of KD is cytotoxic CD8+ T cells. These cells exhibit an aberrantly activated state in the acute phase, with an imbalance between their activation and inhibitory signals (46). Based on the above results, we further speculate that the positive correlation between resting NK cells and CD8+ T cells might indicate their synergistic enhancement of immune responses, which could potentially be related to the initiation and progression of vascular inflammation in KD. In contrast, the negative correlation between neutrophils and CD8+ T cells might suggest a compensatory regulatory mechanism that could be altered in KD, potentially contributing to uncontrolled inflammation. Meanwhile, the bidirectional correlation of CD8+ T cells by DPM2 and DDX59 might represent a key node in immune disorders in KD. We speculate that DPM2 might be positively correlated with CD8+ T cell activation and infiltration, exacerbating vascular inflammation, whereas DDX59 might be negatively correlated with aberrant CD8+ T cell activation, which could potentially influence endothelial integrity. We further hypothesize that imbalanced expression of these two potential biomarkers might be associated with disrupted CD8+ T cell homeostasis and could potentially correlate with coronary artery lesions.

From the perspective of clinical diagnosis and treatment, activation markers of CD8+ T cells hold significant value. Studies have shown that a proportion of peripheral blood CD8 + HLA-DR + T cells exceeding 8.1% can serve as a diagnostic indicator for KD, with a sensitivity of 85.2% and a specificity of 100%. Furthermore, the activation level of CD8+ T cells is closely associated with treatment response: a higher activation level correlates with a greater likelihood of intravenous immunoglobulin (IVIG) resistance (47). Immunological research has also found that CD8+ T cells undergo clonal expansion during the acute phase of KD. These expanded T cell clones gradually disappear after disease remission, strongly suggesting the involvement of antigen-driven specific immune responses in the pathophysiology of KD (48). We hypothesize that antigen-driven specific immune responses might play a role in the pathogenesis of KD, and abnormal activation of CD8+ T cells might be a key factor correlated with vascular inflammation. The activation level of CD8+ T cells has been proposed as a potential marker for evaluating disease severity, therapeutic efficacy, and prognosis in other studies, and our findings offer a computational basis that may guide future research into precise intervention for IVIG-resistant KD patients.

The substance-gene interaction network analysis in this study initially predicted three substances [valproic acid, benzo(a)pyrene, and acetaminophen] that could simultaneously interact with DDX59, RAP2C, and DPM2. Notably, benzo[a]pyrene is a Group 1 carcinogen classified by the International Agency for Research on Cancer (IARC) and a hazardous environmental pollutant with no therapeutic indication; it was therefore excluded from further interpretation. It should be emphasized that the Comparative Toxicogenomics Database (CTD) records both toxicological and therapeutic chemical–gene interactions, and the identified associations are only bioinformatics predictions, with no current clinical or experimental evidence confirming a therapeutic effect in KD. These predicted interactions merely provide preliminary clues for future drug repurposing studies of KD. Regarding valproic acid, literature reports indicate that it is primarily used as an antiepileptic drug and has been confirmed as a safe alternative for children with KD requiring antiepileptic treatment without inducing drug hypersensitivity reactions (49). Regarding acetaminophen, it is commonly used for fever reduction in children, and KD is clinically characterized by poor response to this conventional antipyretic, which serves as a diagnostic clue for differentiating KD from other febrile illnesses (50, 51). In summary, the aforementioned predictive results should be regarded solely as potential clues for future mechanistic studies on the molecular mechanisms of KD, rather than as definitive clinical therapeutic conclusions. Subsequent investigations must validate the effects of the candidate compounds on target gene expression and inflammatory pathways in KD-relevant cellular and animal models. Furthermore, correlative analyses incorporating clinical samples are required to progressively evaluate the potential value of drug repositioning.

This study, through transcriptomic analysis, delved into the molecular mechanisms of KD, discovered and validated the important roles of potential biomarkers RAP2C, DPM2, and DDX59 in disease diagnosis, and revealed the functions of these potential biomarkers in the immune microenvironment and signaling pathways. Despite these encouraging results, further validation via large-cohort studies and in-depth mechanistic research is still needed to translate these findings into clinical applications for KD. This study has several limitations: first, all core conclusions are derived from bioinformatics predictions and correlation analyses. Although the mRNA expression trends were validated by RT-qPCR, the lack of Western blot validation at the protein level precludes confirmation of whether the protein expression of the three candidate genes is consistent with their mRNA levels. Moreover, direct detection of key mitochondrial dynamics proteins (e.g., MFN1, MFN2, DRP1) was not performed. Consequently, all mechanistic inferences regarding the regulation of mitochondrial fission/fusion remain hypotheses extrapolated from the literature and have not been experimentally verified. Second, the RT-qPCR validation included only 5 KD cases and 5 controls. This small sample size results in insufficient statistical power, limits the representativeness of KD clinical heterogeneity, and precludes subgroup analyses based on CAL status or IVIG responsiveness. Third, the bioinformatics analyses relied solely on two datasets from the same platform, lacking external independent validation from multicenter or multi-platform sources. Furthermore, no stratified analyses were conducted for different KD subtypes, thereby restricting the evaluation of the diagnostic value of the potential biomarkers in specific subpopulations.

To address these limitations, future studies should expand the clinical cohort (≥30 cases per group) and simultaneously perform Western blot protein validation and RT-qPCR detection to confirm the expression trends of the three genes and mitochondrial fusion/fission-related proteins. In addition, overexpression/knock down models should be established in human coronary artery endothelial cells and macrophages. These models can be used to directly assess the effects of the candidate genes on mitochondrial dynamics by examining mitochondrial morphology and functional indicators. Furthermore, co-culture systems should be employed to explore the mechanisms by which these genes regulate CD8+ T cell immune regulation. Finally, KD animal models and multicenter independent cohorts are necessary to further evaluate the diagnostic and therapeutic value of these findings. In summary, all conclusions of the present work represent preliminary bioinformatics-based clues that require validation through subsequent experimental and clinical studies.

## Data Availability

The datasets analyzed in this study are publicly available. The training set (GSE73461) and validation set (GSE68004) can be accessed in the Gene Expression Omnibus (GEO) repository (https://www.ncbi.nlm.nih.gov/geo/) under accession numbers GSE73461 and GSE68004, respectively. The mitochondrial dynamic genes (MDGs) were obtained from the MitoCarta database (http://www.broadinstitute.org/mitocarta). The clinical RT-qPCR data are available from the corresponding author upon reasonable request.
